# Numerical Simulation Study on Factors Influencing Anti-Explosion Performance of Steel Structure Protective Doors under Chemical Explosion Conditions

**DOI:** 10.3390/ma15113880

**Published:** 2022-05-29

**Authors:** Haiteng Wang, Zhizhong Li, Yingxiang Wu, Luzhong Shao, Meili Yao, Zhen Liao, Degao Tang

**Affiliations:** 1 State Key Laboratory for Disaster Prevention & Mitigation of Explosion & Impact, Army Engineering University of PLA, Nanjing 210007, China; wht932060322@163.com (H.W.); tdg62@163.com (D.T.); 2 Institude of Defense Engineering, AMS, PLA, Beijing 100085, China; 1224846321@139.com; 3 Northwest Institude of Nuclear Technology, Xi’an 710024, China; ccess027@163.com

**Keywords:** steel structure protective door, explosion impact load, anti-explosion performance, chemical explosion

## Abstract

To study the mechanical deformation characteristics and anti-explosion mechanisms of steel-structure protective doors under chemical explosion shock wave loads, numerical simulations of loads and door damage were carried out using the AUTODYN and LS-DYNA software based on model tuning with actual field test results. The finite element simulation results were compared with the test results to verify the accuracy of the simulation model and material parameters. A parametric analysis was carried out on the influencing factors of the anti-explosion performance of the beam–plate steel structure protective door under typical shock wave loads. The impact of the material strength and geometry of each part of the protective door on its anti-explosion performance was studied. The results showed that the protective door sustained a uniform shock wave load and that increasing the steel strength of the skeleton could significantly reduce the maximum response displacement of the protective door. The steel strength increase of the inner and outer panels had little or a negligible effect on the anti-explosion performance of the protective door. The geometric dimensions of different parts of the protective door had different effects on the anti-explosion performance. Increasing the skeleton height had the most significant effect on the anti-explosion performance. The skeleton’s I-steel flange thickness and the inner and outer panel thicknesses had less significant effects.

## 1. Introduction

The protective doors used in underground engineering need to resist both the positive pressure of explosion shock waves and negative pressures. Protective doors also need to prevent harmful substances from entering the interior structure through the door gap. Protective doors can be classified as steel structures, reinforced concrete structures, ladle concrete structures, or steel-fiber concrete structures based on different materials. The protective door needs to be sufficiently strong structurally to match the strength of the underground structure. The door sash material is often reinforced concrete, which is convenient to produce and has a high stiffness, compression strength, and bending strength. Its corrosion resistance is also satisfactory. Steel has widespread applications. Because steel’s tensile strength is very high, it is suitable for casting and welding. Therefore, it is an ideal material choice for high-strength, large-span protective doors.

Due to the importance of protective doors, many scholars have conducted extensive research on their materials, structural designs, failure modes, and anti-explosion performances under shock waves. Miao et al. [1] used the finite element software ANSYS/LS-DYNA to conduct analysis on the dynamic responses of reinforced concrete protective doors under the shock wave loads of chemical explosions and studied the different effects of shock waves of thermo-pressure bombs and trinitrotoluene (TNT) charges on the response displacements, shear stresses, and failure modes of protective doors. Wang et al. [2] carried out an anti-explosion test of reinforced concrete one-way slabs in the free field when the proportional explosion distance range was 0.15–1 m/kg^1/3^, and they proposed a failure evaluation criterion for one-way slabs based on their test results. Fang et al. [3] used the LS-DYNA finite element analysis software to conduct a comparative study on the anti-explosion capabilities of two types of protective doors (ordinary steel protective doors and protective doors with high-damping rubber filler layers) under typical shock wave loads. Their numerical simulation results showed that using high-damping rubber filler layers in steel protective doors can effectively improve the stress distribution of the door sash panel and avoid the stress concentration phenomena in local areas of the door sash. The door deformation could be reduced by 62% by the rubber filler layers, showing good attenuation resistance to shock waves. Guo et al. [4] used finite element software to establish a model for typical beam–plate steel-structure protective doors and carried out analysis on the factors influencing the anti-explosion performance of the protective door under shock wave loads. Due to the significant stress concentration around the four corners of the doorframe under shock waves, Zhao et al. [5] established a finite element model of the door and the doorframe wall structure based on cantilever beam theory. To effectively reduce the stress concentration, local stress alleviation measures were proposed, which were carried out by establishing artificial weak layers at the doorframe wall and its lining. Li et al. [6] used LS-DYNA to analyze the effect of the explosion shock wave loads of a thermal-pressure bomb on the failure modes of reinforced concrete protective doors. Dong et al. [7] used the ABAQUS finite element software to analyze the dynamic responses of a protective door with a cable and membrane structure under explosion loads and evaluated the anti-explosion performance of the protective door based on the simulation results.

The steel-structure-assembled protective door is composed of various elements, including a skeleton, internal and external panels, a lock head, a reducer, a screw or sleeve combination drive device, sealing beams, filling blocks, and sealing strips. Compared with an ordinary protective door, the structure and connection mode of each component of the protective door are more complex. From the existing research situation, the anti-explosion tests of the protective doors are mostly conducted using reinforced concrete protective doors, and the anti-explosion performance of the beam-plate steel structure protective door is rarely studied. Therefore, in order to study the performance of the steel structure protective door under TNT explosion shock wave load, this paper innovatively carries out numerical simulation analysis on the basis of the anti-explosion test of the beam-plate steel structure protective door. To further examine anti-explosion mechanisms, we investigated the dynamic responses of doors under the shock wave loads of TNT explosions, and the effects of different material strengths and geometries on the anti-explosion performances of the inner and outer panels and skeleton were analyzed.

## 2. Anti Explosion Test Condition of Protective Door

### 2.1. Test Conditions

The field tests of the steel structure protective doors were carried out three times, as well as anti-explosion test research. The test conditions are shown in Table 1. During the test, the spherical TNT bare charge was suspended in the channel preform at a specific distance from the protective door, resulting in the tunnel shock wave load of initiation acting on the door leaf. The tests were carried out in order from small to large. The TNT charges of the three tests were 1.36 kg, 10.2 kg, and 20.0 kg, respectively. The suspension height of the charge center was 0.9 m, and the explosion center was the center of the charge.

### 2.2. Layout of Measuring Points

**Figure 1:**
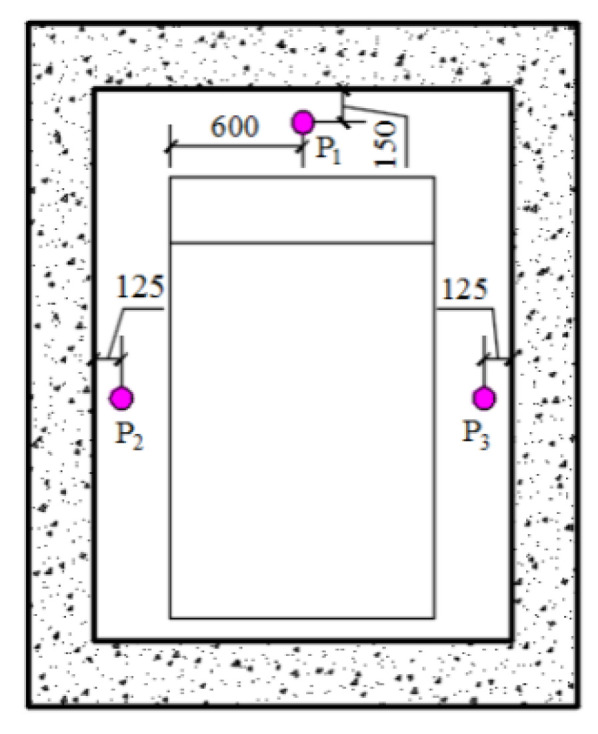
Schematic diagram of pressure sensor layout. Unit: mm.

In the test, the pressure sensor and the acceleration sensor were used to measure the shock wave load and the fan body vibration of the protective door under different loads, respectively. Figure 1 shows the layout diagram of the test pressure sensor. Three solid-state piezoresistive pressure sensors were arranged for each test, which were located directly above the door frame wall (measuring point P1) and on the left and right sides of the door frame wall (measuring points P3 and P2). The acceleration sensor was arranged on the projection surface of the central structural beam of the inner panel of the door leaf, 710 mm away from the top of the protective door.

## 3. Shock Wave Load Analysis for Protective Door

### 3.1. Finite Element Model and Material Parameters

#### 3.1.1. Finite Element Model

**Figure 2:**
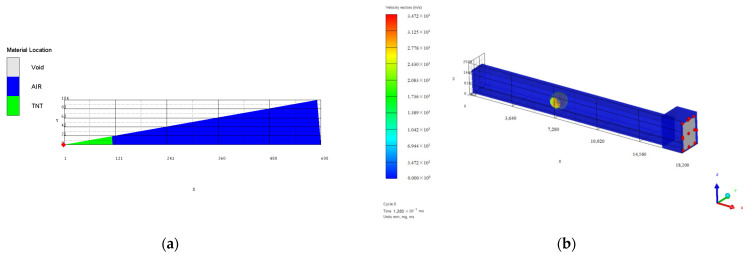
Finite element calculation model of test condition 2: (**a**) 1D model; (**b**) 3D model.

**Figure 3:**
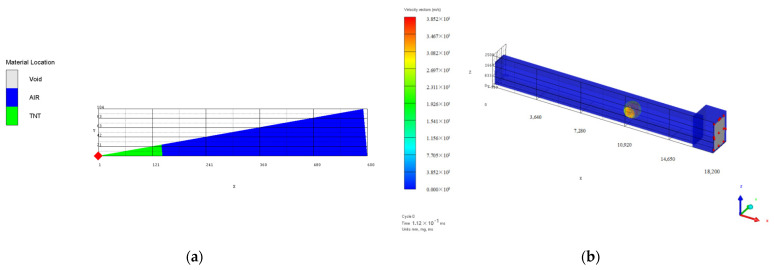
Finite element calculation model of test condition 3: (**a**) 1D model; (**b**) 3D model.

Numerical simulation and analysis were performed on the protective door explosion resistance test conditions conducted above. Due to the small load strength of the first test, the protective door was basically in an elastic deformation state. Therefore, this paper only conducts numerical simulation analysis on test conditions 2 and 3, and the finite element calculation model established by AUTODYN is shown in Figure 2 and Figure 3, respectively. The loading amount of the 2 working conditions was 10.2 kg and 20.0 kg, respectively. The calculations were performed using the Euler 2D multi-material solver and the Euler 3D multi-material solver in the 1D wedge and 3D tunnel finite element models, respectively. The radius of the 1D wedge model was 600 mm, the TNT filling radius was 114.3 mm and 143.0 mm, respectively, and the burst core was the loading center. The coordinate origin of the 3D model is located at the ground midpoint of the tunnel entrance section. The total length is 18.2 m, composed of the tunnel channel of 16 m long, 1.2 m wide, 1.8 m high, and 2.2 m long, with a cross section of 1.9 m wide and 2.5 m high. The protective door is set at the end of the diffusion room with an actual size of 1.4 m, a width of 2.08 m, and a thickness of 0.116 m. For modeling purposes, the corresponding area of the protective gate within 1.4 m, 2.1 m, and 0.1 m is set to unused units to be approximately equivalent to the protective gate. The tunnel port is set to the infinite air domain outside the outflow (flow-out) boundary, and the rest are set to the rigid boundaries to simulate the surrounding wall of the tunnel and the constraint effect of the diffusion chamber wall facing the shock wave. According to the explosion starting position of the test loading, the mapped loading center coordinates in Figure 2b and Figure 3b are (7 m, 0 m, 0.9 m) and (11 m, 0 m, 0.9 m), respectively. The total time was set to 150 ms, and the output time interval was 1 ms and mm-mg-ms.

Aiming at the prominent problem of cell grid size effect in numerical simulation [8,9,10,11], this report presents a comparative analysis of the numerical simulation results of the model under different grid sizes for the same working condition, determining superior cell grid sizes, where the 1D and 3D models are 0.5 mm and 10 cm, respectively.

#### 3.1.2. Mesh Size Effect Analysis

A large number of research results at home and abroad have shown that the finite element numerical calculation results have a greater dependence on the element mesh size. Especially when it comes to the numerical calculation of the explosion shock problem, as the shock wave can be characterized by its extremely short duration and extremely fast energy transmission in the mesh. The mesh size effect of the element is particularly prominent; therefore, for a specific problem, it is necessary to first carry out a mesh size effect analysis to determine a reasonable element mesh size. Theoretically, the smaller the element mesh size is, the higher the calculation accuracy is, but the time cost in actual operation is also an important influencing factor.

**Figure 4:**
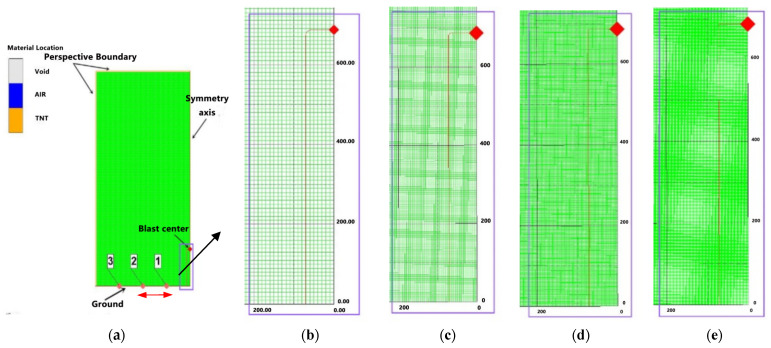
2D finite element model with different element mesh sizes: (**a**) 2D axisymmetric model; (**b**) 10 mm; (**c**) 5 mm; (**d**) 2.5 mm; (**e**) 1 mm.

**Figure 5:**
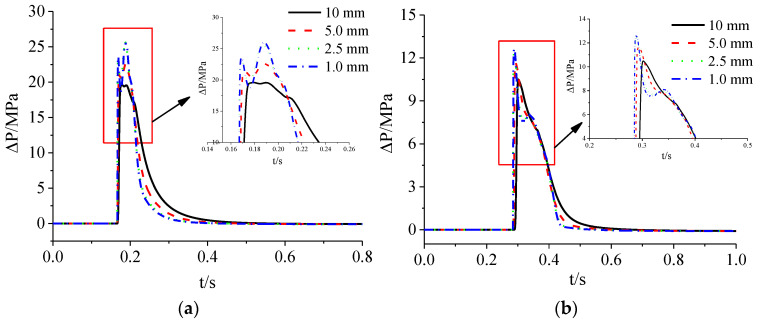

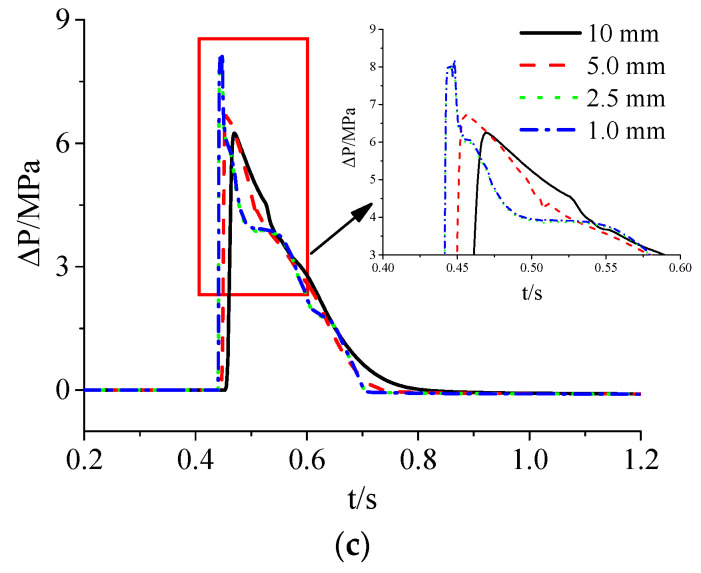
Overpressure time-history curves of measuring points calculated with different mesh sizes: (**a**) x = 0.5 m; (**b**) x = 1.0 m; (**c**) x = 1.5 m.

In order to determine a reasonable element mesh size, this paper selected the columnar TNT charge tunnel explosion test in Test Condition 3 as the research object, and carried out the convergence analysis of the mesh size. Due to the use of Remap technology, the numerical simulation of the columnar charge was carried out in two stages, so its 2D and 3D finite element models were analyzed in turn to determine the optimal element mesh size. Four different element mesh sizes of 10 mm, 5 mm, 2.5 mm, and 1 mm were selected to divide the 2D finite element calculation model, as shown in Figure 4. The size of the model was 400 × 200 cm, and 3 measuring points were set on the ground, and the horizontal distance x from the explosion center was 0.5 m, 1.0 m, and 1.5 m, respectively. The bottom and right boundary of the 2D model were set as rigid boundaries and symmetric boundaries, respectively, and the other boundary was set as a flow-out boundary. Other parameters were set exactly the same. Numerical calculations were carried out for the four finite element models respectively, and the overpressure time-history curves of the corresponding measuring points were extracted, as shown in Figure 5.

As can be seen from Figure 5, the shock wave overpressure peak value at the measuring point increased with the decrease of the cell grid size, and the waveform was also steeper. However, when the cell grid size was reduced from 2.5 mm to 1 mm, the overpressure time-history curves of each measuring point basically coincided. It shows that the influence of the element mesh size on the calculation results gradually converges, and the influence of the smaller mesh model on the calculation results is almost negligible. Therefore, the 2D finite element calculation model in this paper adopted an element mesh size of 2.5 mm.

**Figure 6:**
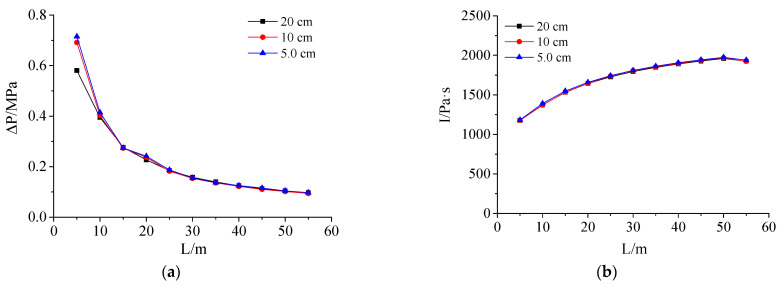
Influence of different mesh sizes on calculation results of tunnel shock wave: (**a**) Influence of mesh size on overpressure peaks; (**b**) Influence of mesh size on impulse.

Similarly, the 3D finite element models of 20 cm, 10 cm, and 5 cm mesh sizes were established, and the calculation results of the 2D model (mesh size 2.5 mm) were mapped to the 3D model for calculation to determine the reasonable mesh size of the 3D finite element model. According to the calculation results, compare the overpressure peak value and the impulse value of the tunnel shock wave at the corresponding measuring points, as shown in Figure 6.

**Figure 7:**
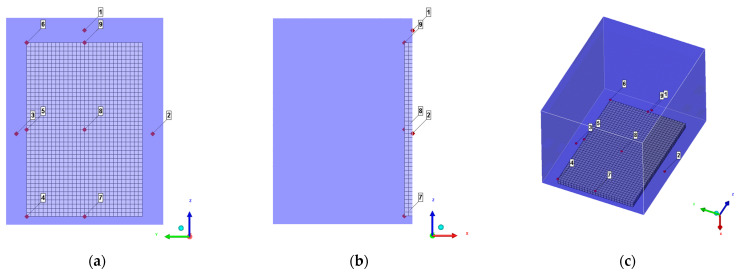
Layout of observation points: (**a**) Front view (X axis); (**b**) Right view (Y axis); (**c**) Oblique axonometric drawing.

**Figure 8:**
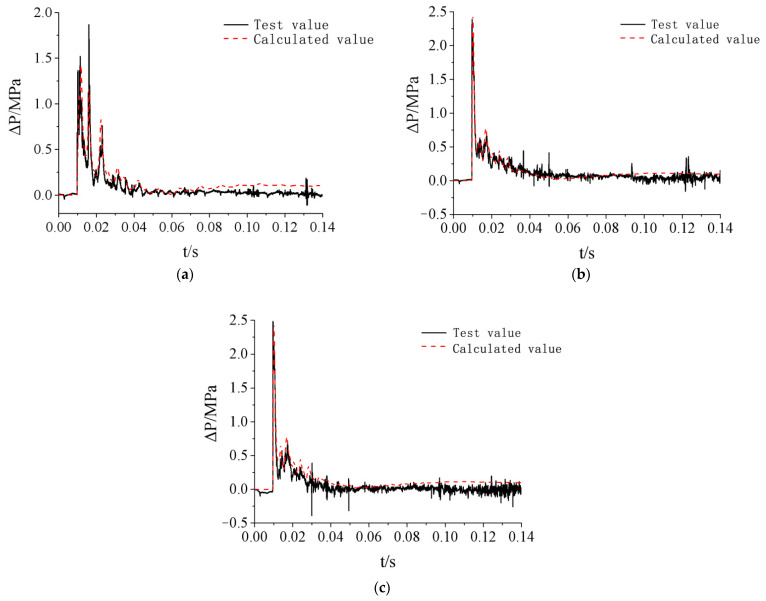
Comparison of overpressure time-history curves of door frame wall measuring points under test condition 2: (**a**) Observation point 1 (Measuring point P1); (**b**) Observation point 2 (Measuring point P3); (**c**) Observation point 3 (Measuring point P2).

**Figure 9:**
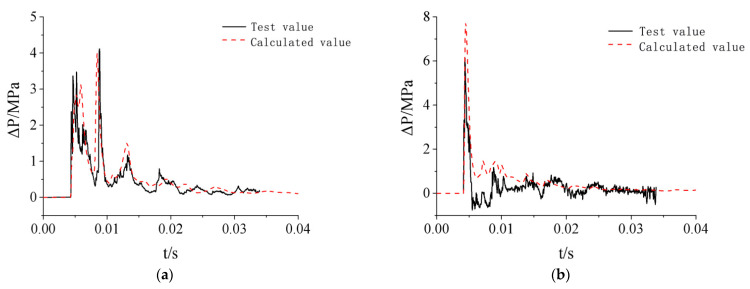
Comparison of overpressure time-history curves of door frame wall measuring points under test condition 3: (**a**) Observation point 1 (Measuring point P1); (**b**) Observation point 2 (Measuring point P3).

**Figure 10:**
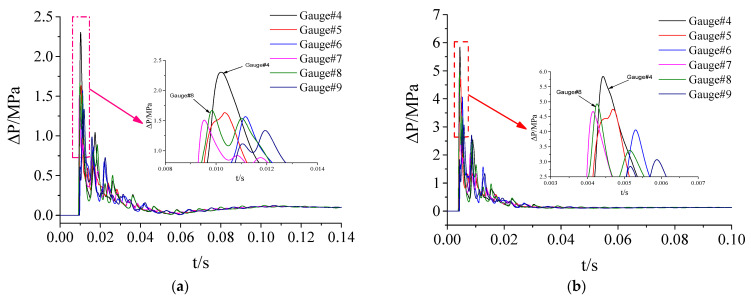
Calculation of overpressure time-history curve of measuring points on the door leaf of protective door: (**a**) Test condition 2; (**b**) Test condition 3.

**Figure 11:**
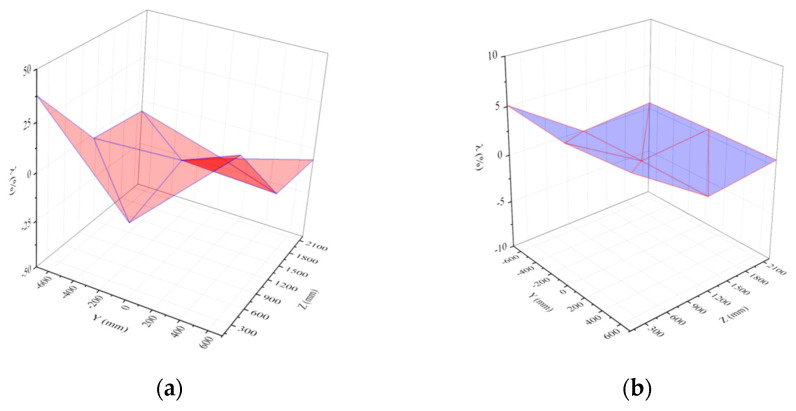
Distribution of shock wave load parameters of protective door under test condition 2: (**a**)Peak overpressure; (**b**) Impulse.

**Figure 12:**
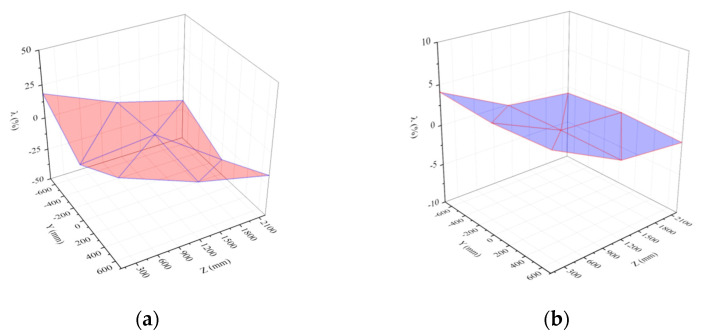
Distribution of shock wave load parameters of protective door under test condition 3: (**a**) Peak overpressure; (**b**) Impulse.

**Figure 13:**
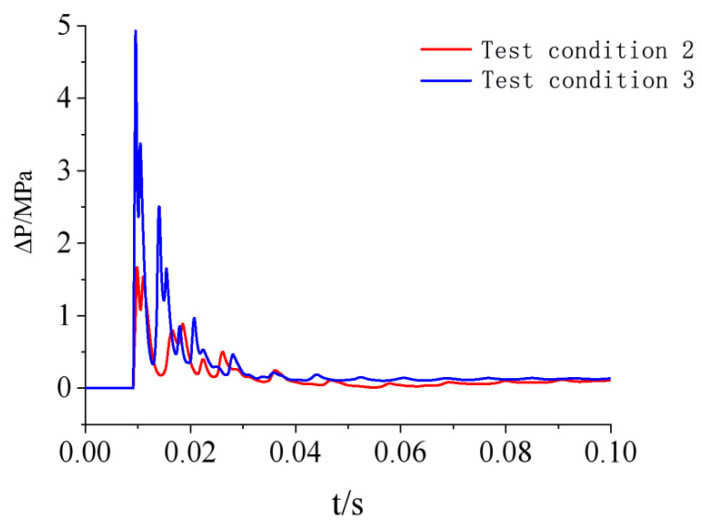
Calculation overpressure time-history curve of protective door.

**Figure 14:**
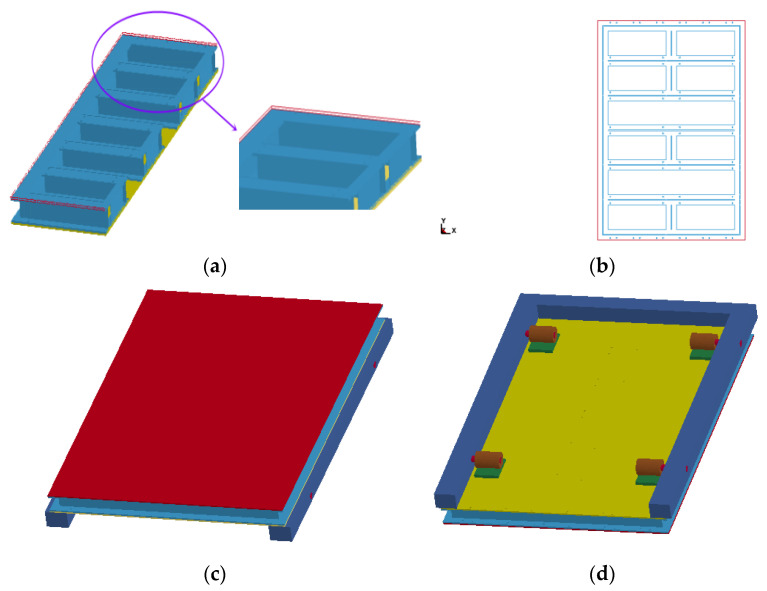
Finite element calculation model of protective door: (**a**) 1/2 finite element model of skeleton; (**b**) Connection mode between skeleton and panel; (**c**) Finite element model of protective door and door frame (front); (**d**) Finite element model of protective door and door frame (back).

**Figure 15:**
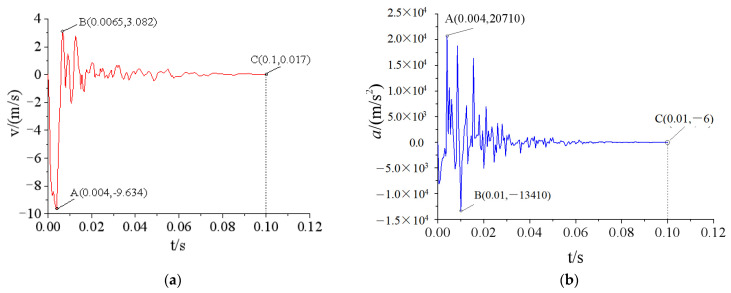
Velocity and acceleration curve of the central node of the blast face of the protective door: (**a**) Velocity curve; (**b**) Acceleration curve.

**Figure 16:**
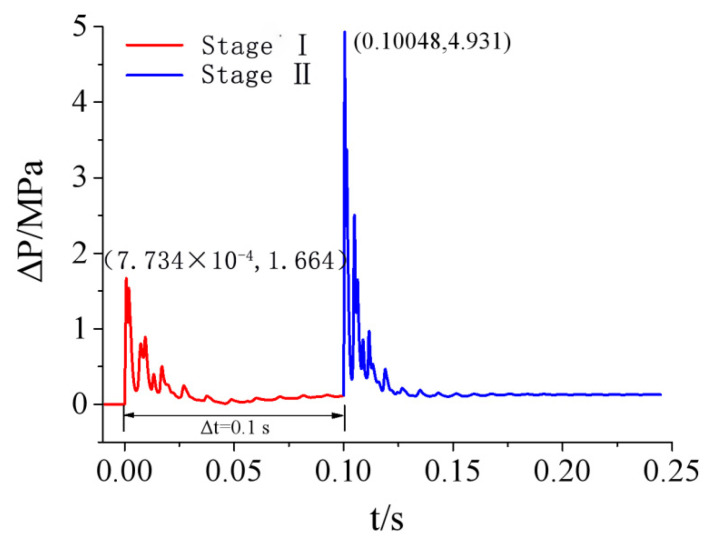
Numerical simulation load of two anti-explosion tests behind the protective door.

It can be seen from Figure 2, Figure 3, Figure 4, Figure 5, Figure 6, Figure 7, Figure 8, Figure 9, Figure 10, Figure 11, Figure 12, Figure 13, Figure 14, Figure 15 and Figure 16 that in the 3D finite element calculation model, the impact of the grid size on the impulse of each measuring point was small, and the shock wave overpressure peak has a relatively greater dependence on the grid size. Especially when the measuring point was close to the tunnel entrance (L < 15 m), the peak overpressure of the tunnel shock wave decreased significantly with the increase of the mesh size. When the grid size was reduced from 10 cm to 5 cm, the calculation results of the two were basically consistent, but the number of grids of the latter calculation model was 2^3^ times that of the former, and its calculation time cost increased significantly. Based on the above analysis, it is reasonable to set the cell mesh size of the 3D model in this paper to 10 cm. This cell size can ensure good calculation accuracy and high calculation efficiency during the analysis process.

#### 3.1.3. Air

The equation of state of the air is approximately described by the ideal gas (Ideal Gas) equation of state, which is obtained based on Boyle’s law and Gai Lusac’s law, and is suitable to describe the state parameters of the changes of various moving gases [12]. The expression is as follows:

(1) $$p=\left(\gamma -1\right)\frac{\rho }{\rho_{0}}E_{0}$$

where: *p* is air pressure; *ρ* is air density after compression or expansion; *ρ*_0_ is initial air density, value 1.225 kg/m^3^; *γ* is adiabatic index, value 1.4; *E*_0_ is the initial specific internal energy of the air, the value is 2.068 e5 J/kg.

#### 3.1.4. Explosives

TNT is described using the JWL equation of state, which is proposed by Lee based on the Jones and Wilkins models, and is suitable to simulate the blast phenomenon of various high explosives and the expansion of the blast products in a specific form as follows [13]:

(2) $$P=A\left(1-\frac{\omega \eta }{R_{1}}\right)e^{-\frac{R_{1}}{\eta }}+B\left(1-\frac{\omega \eta }{R_{2}}\right)\;e^{-\frac{R_{2}}{\eta }}+\omega \rho e$$

where: *P* is the detonation product pressure, Pa; *η* = *ρ*/*ρ*_0_, *ρ* and *ρ*_0_ is the initial density of explosive and the density of detonation products, kg/m^3^; *e* is the initial internal energy per unit mass explosive, J/kg; *A*, *B*, *R*_1_, *R*_2_ and *ω* are the five constants of the equation of state that need to be determined by cylinder test and two-dimensional hydrodynamic program [14,15]. The equation of state parameters of TNT explosives are selected, as shown in Table 2. The dominant pressure term in the equation of state changes at different stages of the loading explosion. Three terms in the right end of the upper formula (From left to right, it is represented by PH, PM and PL) play the main role in the high, medium, and low pressure ranges, respectively, while in the late stage of the detonation product expansion, the pressure of the detonation product is mainly determined by PL, the first two roles are negligible.

### 3.2. Layout of the Observation Points

In order to compare the calculated value of the shock wave load on the frame wall with the test value and analyze the shock wave pressure distribution characteristics on the protective door fan, 9 observation points are arranged on the frame wall and protection surface, as shown in Figure 7. Among them, the three observation points on the door frame wall correspond to the three pressure sensors in the test, and the six observation points on the protective door are 700 mm and 1050 mm apart in horizontal and vertical directions, respectively. Table 3 shows the observation point numbers and the corresponding position coordinates.

### 3.3. Comparison of Numerical Simulations and Experimental Results

#### Overpressure Time-History Curves of Observation Point on Doorframe Wall

The simulated overpressure time-history curves of three observation points on the doorframe wall were extracted and compared with measured data, as shown in Figure 8 and Figure 9. Since the arrival time of the chemical explosion shock wave load is not related to the failure effect analysis of the protective door, to intuitively compare the difference in the shock wave load between the simulation and experimental data, the simulated time-history curves and the measured curves were aligned to have the same arrival time of the shock wave. The differences are shown in Table 4. The peak value of the first wave on each curve was selected to compare the overpressures.

Figure 8 and Figure 9 and Table 4 show that the simulated overpressure curves of the observation point on the doorframe wall were in good agreement with the measured curves. The simulated waveform exhibited the same oscillation characteristics as the measured curve. The errors between simulation and testing were basically within 10%. The measured overpressure curve of P3 showed significant drift toward the negative direction of the coordinate axis in test condition 3, and the measured shock wave parameters at this point were small. Therefore, the data of P3 were discarded (Figure 9b).

The above analysis indicates that the simulation model established in this study based on the AUTODYN finite element analysis software can accurately predict the explosion shock wave loads on protective doors.

### 3.4. Calculation Load on Protective Door

To further analyze the distribution characteristics of explosion shock wave loads on the protective door under different test conditions and calculate the load on the protective door, the time-history simulation curves of the overpressure at six observation points on the door sash under two conditions were analyzed, as shown in Figure 10.

As can be seen from Figure 10, the shock wave load on the door sash was similar to that on the doorframe wall in terms of the load pattern. Due to the interactions between the diffusion chamber wall and the shock wave, the shock wave load acting on the protective door had four or five distinct peaks, and the load levels at different positions on the door sash were different. Among the six typical observation points selected, No. 4 at the lower left corner of the door sash had the maximum overpressure, while No. 9 at the center of the upper end of the door sash had the minimum overpressure. The simulated peak overpressure and impulse data at each observation point are shown in Table 5.

To analyze the shock wave load distributions at different positions on the door more intuitively, the data of observation point No. 8 at the center of the door were set as reference values. The ratio *Φ* is defined as follows:

(3) $$\phi =\frac{F_{n}}{F_{8}}$$

where *F*_n_ and *F*_8_ are the peak overpressure or impulse at observation points No. n and No. 8, respectively.

Figure 11 and Figure 12 show the differences in the shock wave load parameters between the two test conditions. Table 5 and Figure 11 and Figure 12 show that the shock wave loads on the door sash were nearly the same except at observation points No. 4 and No. 9, which had larger simulation values. Compared with the reference data of observation point No. 8, the deviations from the peak overpressures at other observation points were less than 17.6%, and the deviations from the impulse were less than 5.1%.

According to the above analysis, the test in this study did not generate a uniform shock wave load distribution on the door sash, and the load near the door sash edge was significantly different from the loads at other positions. However, the load variation in the central area of the door sash was very small, and this area occupied a large proportion of the total door sash area. There was a 10-cm lap width between the right or left side of the door and the doorframe wall. There was an 8-cm lap width between the upper side of the door and the doorframe wall. In the lap width area, the force acting on the door sash was directly transmitted to the doorframe wall. Therefore, the uneven load distribution in the lap width area had little or negligible impact on the dynamic response of the door. Based on this analysis, the shock wave load acting on the door sash was simplified to a uniform distribution in this study, and the simulated load at observation point No. 8 in the center of the door was used as the uniformly distributed load. The simulated overpressure curves of the two test conditions are shown in Figure 13.

## 4. Failure Effect Analysis of Steel Structure Protective Door

### 4.1. Finite Element Model and Material Parameters of Steel Structure Protective Door

#### 4.1.1. Finite Element Model

The test object was a single entrance/exit protective sealing door ZCFM1220(5) of civil air resistance level 5, and a size of 1.4 × 2.08 m. The door was composed of a skeleton, internal and external panels, a lock head, a reducer, a screw or sleeve combined drive device, a sealing beam, filling blocks, and sealing strips. The door material was ordinary carbon steel Q235A, and the door skeleton profile was HW100 section steel. The skeleton was surrounded by a complete circle of H-beam. The transverse arrangement length of the main beam was 1200 mm (equal to the net width of the door opening). The secondary beam was segmented and fixed with the main beam and the side frame and the panel by 4.8 grade M10 × 1.5 high strength bolts, and the panel was made of 8 mm thick ordinary hot-rolled steel plate. There were 4 Φ40 lock heads distributed on the inner surface of the door, made of 45# steel, one set on the left and one set on the right, which were driven by the reducer and the combination of screw and sleeve through the connecting plate. The sealing beam at the threshold was composed of L50 × 32 angles with adhesive strips inside. The driving mechanism was similar to that of the lock head, and the extrusion force in the direction of the door sash was reversely borne by three convex handles fixed on the inner surface of the door sash. The sealing joints at the corners on both sides were arranged on the door frame, and the trapezoidal frame was filled with sealing blocks. The Ω rubber strip was set on the circumference of the inner surface of the door (except the bottom). The pressure strip plate was an ordinary 8 mm thick steel plate, and the pressure bolt was an ordinary bolt M6 × 1, tapped through the panel and profile flange with a spacing of 250 mm. The door frame adopted an L125 equilateral angle, split design, and the corner end was connected with an L160 equilateral angle, bolt sub, and positioning pin assembly, and the screw anchor bolts were used to replace the welded anchor hooks. Compared with the common protective door, the structure and connection mode of each component of the test door is more complex.

To facilitate the analysis, the components and connection modes of the test door were simplified in the finite element modeling. Specifically, the dynamic responses of the main stressed components were considered, including the skeleton, panels, and screw or sleeve combined drive device, while the reducer, sealing strips, and sealing beams were ignored. The connection mode of using high-strength bolts between the main beam and the secondary beam or the skeleton was simplified to a consolidation connection at the components. The connection mode of using high-strength bolts between the skeleton and the panels was simplified to a consolidation connection at the bolts. The circular bolt hole with threads was simplified as a square hole.

A finite element model of the steel structure protective door was established according to the aforementioned method, with the dimensions of all the parts the same as the actual sizes, as shown in Figure 14. Figure 14a shows the finite element model of half of the skeleton. The coordinate origin overlapped with the vertex at the lower left corner of the inner panel of the protective door. The square hole in Figure 14b is the consolidation area between the skeleton and the inner and outer panels. The side length of the hole was 10 mm, and the other parts were set for automatic surface-to-surface contact. Figure 14c,d show the finite element models of the door and the doorframe, respectively. Four rectangular steel gasket plates, each with a thickness of 2 cm, had fixed connections with the inner panel. The four sleeves also had fixed connections with their gasket panels. The four screws and their corresponding sleeves had automatic surface-to-surface contact. The screws and their corresponding holes in the doorframe also had automatic surface-to-surface contact, as did the connection between the door and the doorframe. This type of contact is defined by the keyword “*CONTACT_AUTOMATIC_SURFACE_TO_SURFACE” [16]. Both the static and dynamic friction coefficients were set to 0.3. Default values were used for other contact parameters. Each element in the finite element model was modeled by the SOLID164 hexahedral constant stress element with a Lagrangian grid. To ensure regular meshing of the cell grid, different grid partition methods were used for the components with different shapes. The “mapped” grid partition method was adopted for the skeleton, panels, and gasket plate with an element size of 5 mm. There were 334,036 elements in the skeleton, 232,960 elements in the inner and outer panels, and 8640 elements in the gasket plate. The “sweep” grid partition method was adopted for the cylindrical screws, sleeves, and doorframe, with an element size of 10 mm. There were 3596 elements in the screws, 7920 elements in the sleeves, and 69,360 elements in the doorframe.

Because there was no evident deformation or damage to the doorframe during the test, the impact of deformation on the analysis results can be ignored. The doorframe wall was simplified to a rigid body. The three translational and three rotational degrees of freedom were constrained for the doorframe wall to ensure that the doorframe did not move or rotate during the deformation process of the door.

#### 4.1.2. Steel

The material of the skeleton, panels, and gasket plate of the door was Q235 steel. The material of the screws and sleeves was No. 45 steel. The keyword “*MAT_PLASTIC_KINEMATIC” in LS-DYNA was used to describe the mechanical properties of the steel material under the shock wave load. This material model was a hybrid model of isotropic hardening and follow-up hardening with the effect of the strain rate [17]. The Cowper–Symonds equation [18] was used to calculate the strain rate effect of steel. The dynamic yield strength is calculated as follows:

(4) $$\sigma_{y}=\left[1+{\left(\frac{\dot{\varepsilon }}{C}\right)}^{\frac{1}{P}}\right]\left(\sigma_{0}+\beta E_{p}\varepsilon_{p}^{eff}\right)$$

where *σ_y_* is the dynamic yield stress, *σ*_0_ is the initial yield stress, $\dot{\varepsilon }$ is the strain rate, *C* and *P* are strain rate parameters, $\varepsilon_{p}^{eff}$ is the effective plastic strain, *β* is the hardening parameter, and *E_p_* is the plastic hardening modulus. The values of *C* and *P* are related to the steel type. Table 6 shows the strain rate data of different types of steel. The *C* and *P* values used in this paper were 40.4 s^−1^ and 5, respectively.

#### 4.1.3. Doorframe

The doorframe was a prefabricated part made of C40 concrete. It was approximated as a rigid body in the simulation model. The keyword “*MAT_RIGID” [17] in LS-DYNA was used in this study to define the doorframe. The use of this keyword only required data to be input for the material density, elastic modulus, and Poisson’s ratio. To avoid convergence problems in the numerical simulations, these data in the model should match the data of real materials. The material parameters of the protective door’s finite element model are summarized in Table 7.

### 4.2. Shock Wave Loading Pattern

The numerical simulation results in Section 3.4 show the overpressure time-history curves of the protective door under test conditions 2 and 3 (Figure 13). Because the same door was used in all three tests, the door damage conditions revealed that the impact of the first explosion test could be neglected, but the damaging impacts of the second and third tests were more severe. The door experienced deformation in the second test. To fully consider the impact of the second test on the final damage, the restart analysis function of LS-DYNA was used to analyze test conditions 2 and 3.

In the restart analysis, the response of the door under the previous load (stage I) was used as the initial condition of the second load (stage II). Therefore, the simulation result of stage I had a significant impact on stage II. Theoretically, if the simulation time of stage I was longer, the structural response could better match the real situation. However, compared with the general dynamic load, the explosive load had a much shorter acting time. Therefore, the structure could be restored to a quasi-static state in a shorter period of time. Further increasing the simulation time of stage I had little impact on the calculation result, wasting computing resources and time. To determine a reasonable simulation time for stage I, the dynamic response of the protective door in stage I under test condition 2 was first analyzed. Figure 15 shows the time-history curves of the velocity and acceleration of the central node of the protective door at t = 0.1 s.

Figure 15 shows that the velocity and acceleration curves experienced high-frequency oscillations with large amplitudes and then recovered to a static state. The oscillation frequency of the acceleration curve was greater than that of the velocity curve due to the characteristics of the explosive load and the natural frequency of the protective door. Figure 15a shows that the maximum velocity (v = −9.6 m/s) of the door occurred along the loading direction at t = 0.04 s, and the maximum velocity in the reverse direction (v = 3.1 m/s) occurred at t = 0.065 s. Then, the velocity diminished gradually after several oscillations. Similarly, the acceleration curve in Figure 15b shows that the maximum acceleration in two directions occurred at t = 0.004 and 0.01 s, respectively, and approached a horizontal straight line after t = 0.06 s.

Ref. [23] reported numerical simulation results of a restart analysis on the dynamic responses of reinforced concrete columns under repeated explosive loads, and it was suggested that when the velocities of all the nodes on the structure were less than 0.1 m/s, the structure could be regarded as having reached static equilibrium. Thus, the calculation at this stage could be terminated. Figure 15 shows that the velocity of the central node of the door was 0.017 m/s (<0.1 m/s) at t = 0.1 s, indicating that the structure had basically recovered to static equilibrium, and further increasing the computing time would not change the simulation result. Therefore, the numerical simulation of stage I was terminated at t = 0.1 s and the door deformation and response parameters at this moment were used as the initial conditions for the analysis in the next step. Figure 16 shows the time-history curves of the load simulation of the last two tests.

### 4.3. Comparison between Simulation and Measurements

#### 4.3.1. Comparative Analysis of Displacement

**Figure 17:**
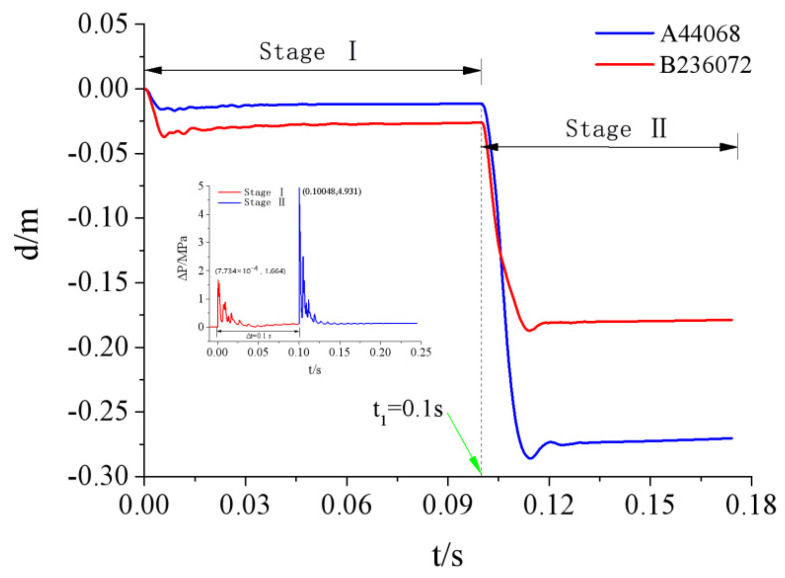
Displacement time-history curve of typical nodes of protective door.

Figure 17 shows the simulated displacement curves of two typical nodes on the door under explosive shock wave loads. Node A was numbered 44,068 with coordinates (0.7 m, 0 m, 0.116 m), located at the midpoint of the lower edge of the outer panel. Node B was numbered 236,072 with coordinates (0.7 m, 1.04 m, 0.116 m), located at the center of the outer panel.10^−4^

Figure 17 shows that under the two shock wave loads with peak overpressures of 1.66 and 4.93 MPa, the displacement curve can be divided into two different stages with t_1_ = 0.1 s as the dividing line. The displacement curve of a given node has similar variation processes in different stages, i.e., reaching the peak displacement rapidly under the load and then reaching a stable residual deformation condition after a process of small oscillations and rebounds. A larger peak overpressure resulted in a shorter oscillation period. A smaller displacement rebound resulted in a larger residual displacement. In stage I, the displacement rebounds of these two nodes were approximately 30.0%, while they were only approximately 5% in stage II. This was because the peak load in stage II was greater, and the door had already sustained damage in stage I. Therefore, the deformation recovery capability of the door in stage II was significantly less than that in stage I, resulting in a larger residual deformation.

The relative displacements of the two typical nodes changed significantly in the different deformation stages. In stage I, the maximum displacement of the door was 3.7 cm, appearing at node A in the center of the outer panel. The maximum displacement of node B at the midpoint of the lower edge of the outer panel was 1.7 cm, which was only 45.9% of the displacement of node A. In stage II, the maximum displacement was 28.6 cm, appearing at node B, while the displacement at the center of the outer panel was only 65.4% of the displacement of node B. These phenomena were due to the load level and the boundary conditions of the door. Because the door was supported by the doorframe walls on the two sides and the upper part, and as well as the lower part’s similarity to a free edge, the displacement of the midpoint of the lower edge of the outer panel was greater than the displacement of the center of the panel when the door was damaged. These simulation findings were consistent with the experimental results.

#### 4.3.2. Comparative Analysis of Failure Modes

**Figure 18:**
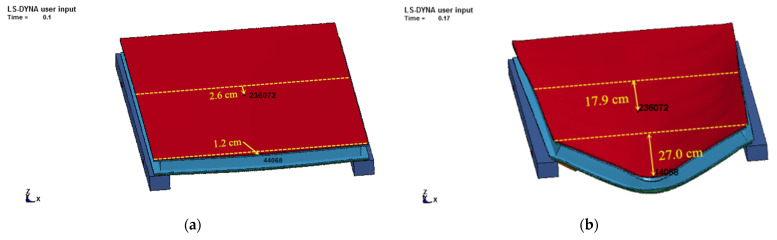
The failure pattern of the two anti-explosion tests of the protective door: (**a**) Stage I; (**b**) Stage II.

The simulated failure pattern diagrams of the two tests are shown in Figure 18, which directly reflect the final deformation condition and failure characteristics of the protective door under shock wave loading.

As can be seen from Figure 18, the door deformation was small after the stage I test without evident damage to the door sash. However, the door sash was significantly deformed and warped with inward bulging after the stage II test. The bulging became more significant in the lower part of the door. These simulation results were nearly the same as the experimental observations of the failure modes. The simulation results showed that the maximum residual deformation was 27.0 cm. The experimental results showed that the maximum residual deformation was 25.0 cm. The difference was only 8%, indicating good agreement.

The above analysis showed that the simulation model established in this study can accurately simulate door deformation and damage under explosive loads in a tunnel. It also indicates the necessity of model simplifications for the analysis of the protective door.

## 5. Analysis on Influence Factors of Anti-Explosion Performance of Protective Door

The beam–plate steel structure protective door is a composite type of door that has an I-steel skeleton and panels. Its anti-explosion performance is affected by the material strength and geometry of each component. In this section, the validated finite element simulation model results are used to further analyze the effects of the influence factors on the anti-explosion performance.

### 5.1. Overview of Finite Element Analysis

**Figure 19:**
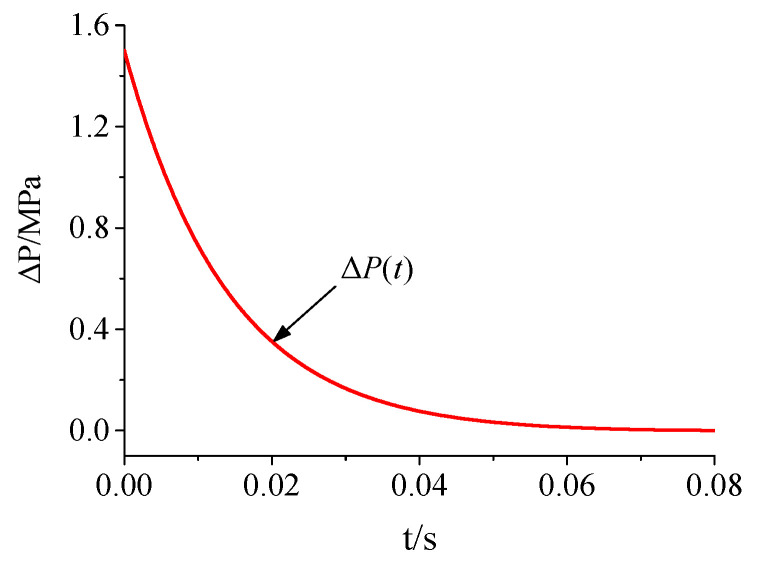
The overpressure time-history curve of the load.

A typical explosive shock wave load was selected with a peak overpressure $\Delta P_{m}$ of 1.5 MPa. The acting time of the positive pressure was 80 ms. The overpressure time-history curve of the load is shown in Figure 19.

**Figure 20:**
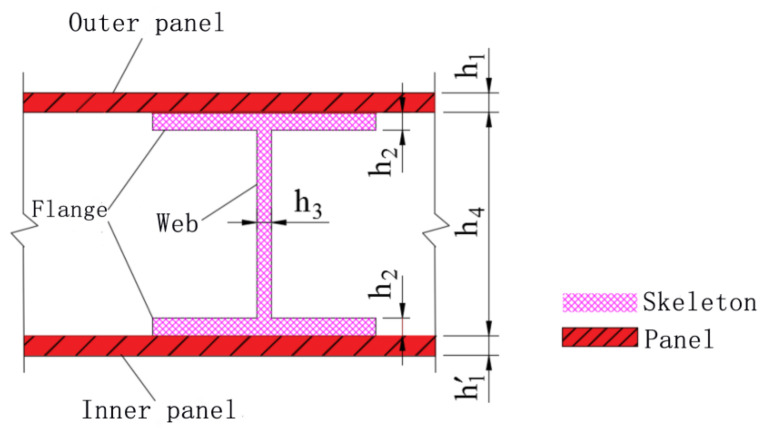
Schematic diagram of cross section of each component of door sash.

Figure 20 shows the cross-section of each component of the door sash for the convenience of analysis. $h_{1}$ is the outer panel thickness, ${h^{\prime }}_{1}^{}$ is the inner panel thickness, $h_{2}$ is the I-steel flange thickness, $h_{3}$ is the I-steel web thickness, and $h_{4}$ is the skeleton height. In the numerical simulation, only the steel strength of each component of the door sash or the cross-sectional dimensions of the different components were varied. The overall geometric dimensions of the door sash and the relative positions of the components remained unchanged, and they were the same as the parameters used in the simulation model shown in Section 4.1. Different finite element models were obtained quickly by using the APDL parametric modeling method in ANSYS.

### 5.2. Effect of Steel Strength

To analyze the effect of the material strength of each component on the dynamic response of the door, three commonly used steel materials, Q235, Q275, and Q345, were selected for analysis. The working conditions are shown in Table 7. The cross-sectional dimensions of each component of the door sash were the same ($h_{1}={h^{\prime }}_{1}=h_{2}=$ 8 mm, $h_{3}=$ 6 mm, and $h_{4}=$ 100 mm). Figure 18 shows the simulation results of the displacement at the center of the door.

**Figure 21:**
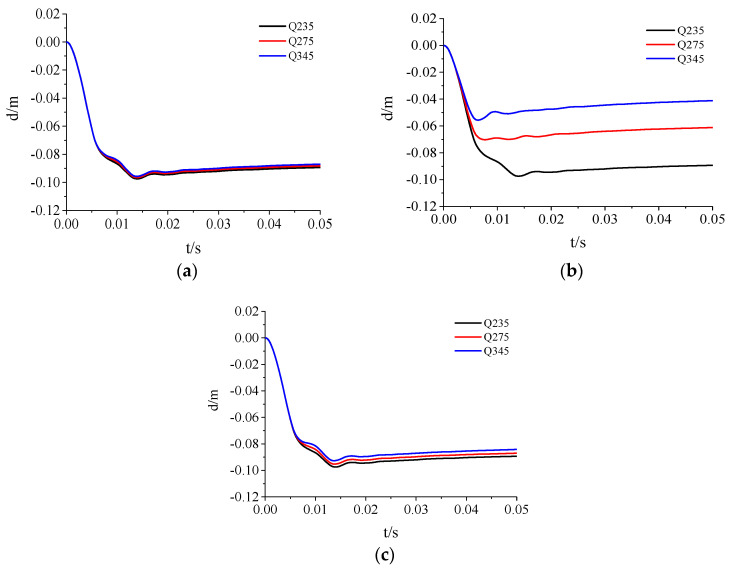
Displacement time-history curves of protective doors with different material strengths: (**a**) Outer panel; (**b**) Skeleton; (**c**) Inner panel.

**Figure 22:**
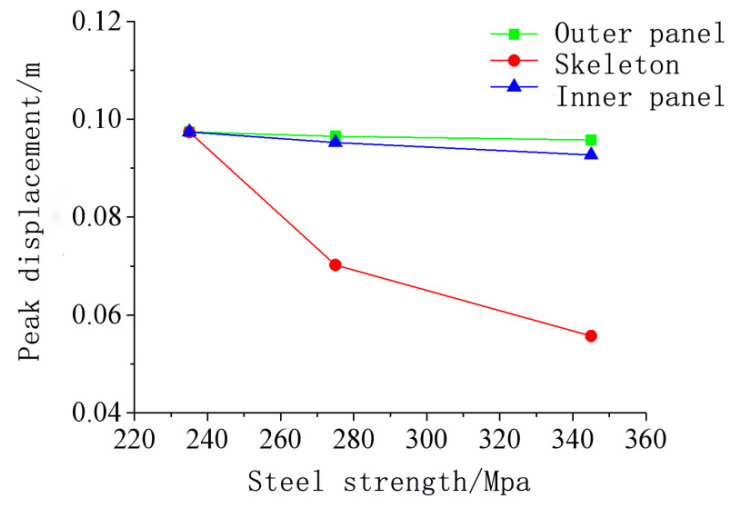
Relationship between the peak displacement of the protective door and the steel strength.

**Figure 23:**
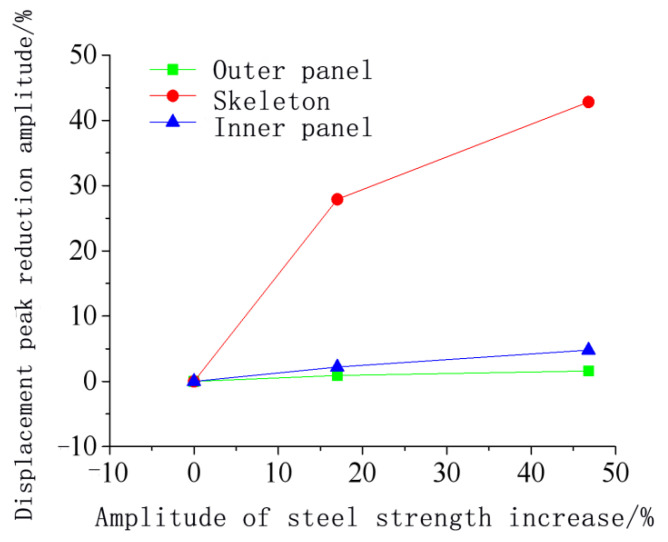
Relationship between the decreasing amplitude of the displacement peak value of the protective door and the increasing amplitude of the steel strength.

Figure 21 shows that as the steel strength increased, the peak displacement at the center of the door gradually decreased, but the reduction was closely related to the components of the door sash. The Q235 steel data were used as a reference for comparison. Figure 22 and Figure 23 show the relationship between the peak displacement and the component strength. The peak displacements under different loading conditions are shown in Table 8.

Figure 23 shows that the steel strength variations resulted in different anti-explosion performances for different parts of the door. The order of the deformation reduction from large to small was the skeleton, the inner panel, and the outer panel. The increase in the steel strength of the inner and outer panels had an insignificant effect on the door sash deformation. The simulation showed that when the steel strength of the inner and outer panels increased by 42.8%, the maximum displacement at the door center decreased by only 1.6% and 4.8%, respectively. Compared to the inner and outer panels, increasing the skeleton strength could significantly reduce the displacement at the door center. When the steel strength of the skeleton increased by 17.0% and 46.8%, the maximum displacement at the door center decreased by 27.9% and 42.8%, respectively, indicating that the anti-explosion performance increased significantly.

### 5.3. Effect of Geometric Dimensions

To systematically study the effects of the cross-sectional geometric dimensions of each component of the steel structure protective door on the dynamic response of the door sash under the explosive load, the following parameters were studied by the numerical simulations: (1) the outer panels, inner panels, skeleton I-steel flanges, and skeleton I-steel webs with different thicknesses, and (2) skeletons with different heights. The steel was Q235. The one-factor-at-a-time approach was used in the simulation to analyze the effects of these parameters. The working conditions are shown in Table 9, where condition 2 was set as a reference for comparison.

**Figure 24:**
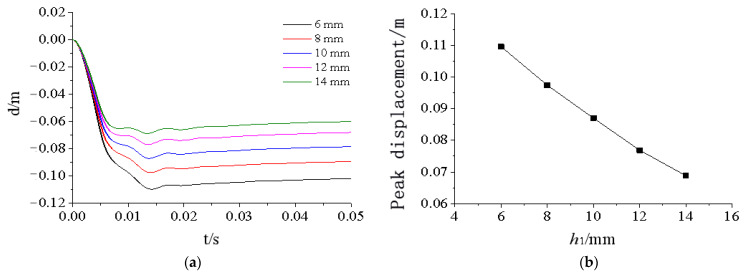
Influence of outer panel thickness $h_{1}$ on displacement response of protective door: (**a**) Displacement time-history curve; (**b**) Relationship between peak displacement and thickness of outer panel.

**Figure 25:**
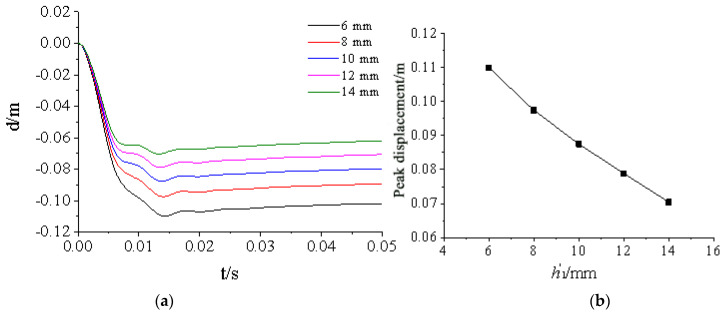
Influence of inner panel thickness ${h^{\prime }}_{1}$ on displacement response of protective door: (**a**) Displacement time-history curve; (**b**) Relationship between peak displacement and inner panel thickness.

**Figure 26:**
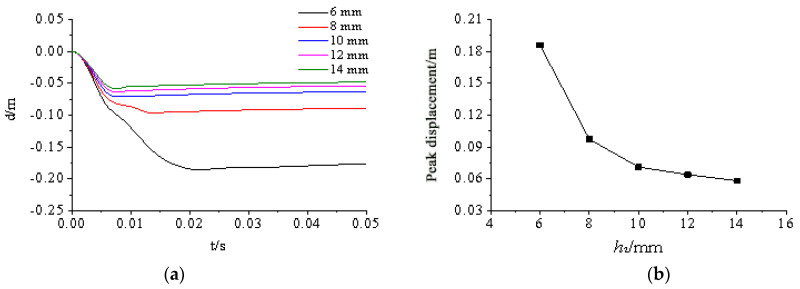
Influence of flange thickness $h_{2}$ on displacement response of protective door: (**a**) Displacement time-history curve; (**b**) Relationship between peak displacement and flange thickness.

**Figure 27:**
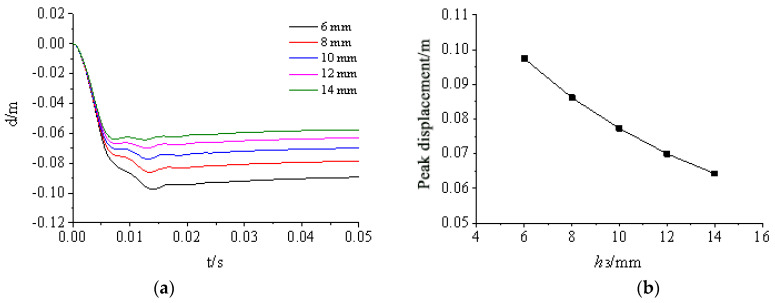
Influence of web thickness $h_{3}$ on displacement response of protective door: (**a**) Displacement time-history curve; (**b**) Relationship between peak displacement and web thickness.

**Figure 28:**
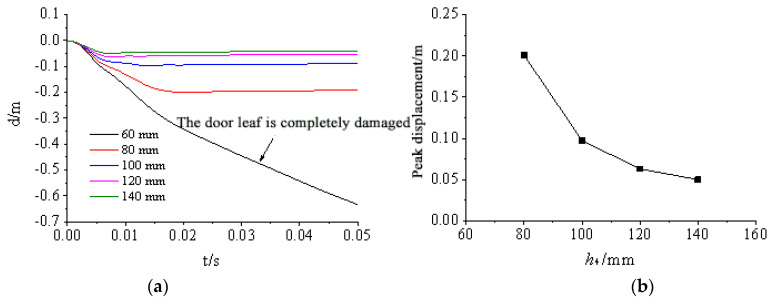
Influence of skeleton height $h_{4}$ on displacement response of protective door: (**a**) Displacement time-history curve; (**b**) Relationship between displacement peak and skeleton height.

Figure 24, Figure 25, Figure 26, Figure 27 and Figure 28 show the simulated displacement curves at the door center, along with the relationship between the peak displacement and the geometric dimensions.

**Figure 29:**
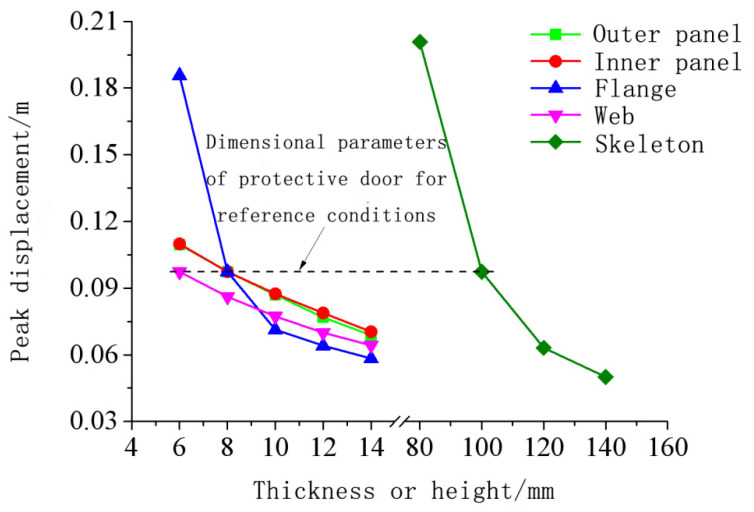
Influence of geometric dimensions on the deformation of protective doors.

**Figure 30:**
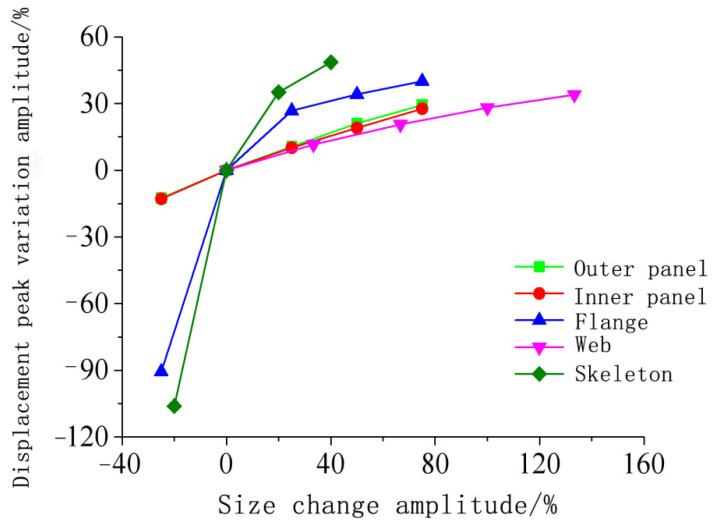
Relationship between the maximum displacement reduction and the geometric size increase.

When the component thickness or height increased, the peak displacement decreased, but the variation trends were different for different components. The maximum displacement decreased linearly when the thickness increased for the inner panels, outer panels, or skeleton I-steel web. For the skeleton I-steel flange and the skeleton, the peak displacement decreased rapidly at first and then slowly when the flange thickness or the skeleton height increased, with a concave curve shape (Figure 26b and Figure 28b). Figure 29 shows the variations of the peak displacements under the influence of the geometric dimensions of each cross section. Figure 30 shows the relationship between the change in the peak displacement and the change in the geometric dimensions. The horizontal axis in Figure 30 shows the dimension changes, with a positive value representing an increase and a negative value representing a decrease. The vertical axis in Figure 30 is the change in the peak displacement, with a positive value indicating that the peak displacement was less than that of reference condition 2 and a negative value indicating that the peak displacement was greater than that of reference condition 2.

Figure 30 shows that increasing the skeleton height was the most effective method in improving the anti-explosion performance. Other effective factors were the skeleton I-steel flange thickness, the outer panel thickness, and the inner panel thickness, in the order from more significant to less significant. The factor that had the weakest effect was the I-steel web thickness. The change in the peak displacement at the door center was linearly proportional to the changes in the thicknesses of the inner panel, outer panel, and I-steel web. The curves of the peak displacement change of the inner and outer panels almost overlapped, indicating that increasing the thicknesses of the inner and outer panels had essentially the same effect on the anti-explosion performance of the protective door.

## 6. Conclusions

This study mainly addresses the effects of design parameters on the damage of the protective door under TNT chemical explosive shock wave loads. Two field test conditions were simulated for the steel structure protective door. The numerical simulation model was established for a typical beam–plate steel structure protective door. Dynamic and finite element simulations were conducted to examine the explosive shock wave loads and the dynamic response of the door. The conclusions were as follows:

(1) Based on finite element simulations, AUTODYN and LS-DYNA were used to simulate the explosive shock wave loads and the damage to the protective door, respectively. First, AUTODYN finite element analysis was conducted to calculate the explosive shock wave loads in the tunnel with a TNT charge. The calculated pressure values of the doorframe wall were compared with the measured results, showing good agreement. Based on the simulation results of the shock wave load distribution characteristics on the door sash, a reasonable load on the door was determined. Then, the LS-DYNA finite element software was used to establish a model of the protective door. The damage effects of the two anti-explosion tests were simulated by using the restart analysis function. The restart conditions and the load form were determined by trial simulations. The simulation results of the door damage and the residual displacement were compared with test data, and the finite element model was validated.

(2) The shock wave load acting on the door sash had the same form as the load acting on the measurement position on the doorframe wall. Both forms exhibited distinct multi-peak patterns. The shock wave parameters near the edge of the door sash were significantly different from those at other parts of the door sash. Overall, the load distribution on the protective door can be regarded as a uniform shock wave load distribution. The local support of the skeleton to the inner and outer panels led to significant differences in the stiffness of the door sash at different positions, resulting in an uneven stress distribution on the panel. When the load increased, the connecting area of the door was destroyed first, and the maximum displacement on the door sash gradually shifted from the middle to the bottom. An effect of the free boundary condition at the lower edge of the door sash on the displacement distribution was observed.

(3) Increasing the steel strength of the skeleton could significantly reduce the maximum response displacement of the protective door. When the steel strength of the skeleton increased by 17.0% and 46.8%, the maximum displacement decreased by 27.9% and 42.8%, respectively, indicating that the anti-explosion performance increased significantly. The steel strength increase in the inner or outer panel had little or a negligible effect on the anti-explosion performance of the protective door.

(4) The geometric dimensions of the different components of the protective door had different effects on the anti-explosion performance. Increasing the skeleton height was most effective for improving the anti-explosion performance. Increasing the thickness of the skeleton I-steel flange and the inner or outer panel led to a smaller improvement in anti-explosion performance. The peak displacement of the protective door decreased rapidly first and then slowly with an increase in the thickness of the I-steel flange or the skeleton height, while the peak displacement decreased approximately linearly with the increase in the thickness of the inner or outer panel or the I-steel web.

## Figures and Tables

**Table 1:** Test condition table.

Test Conditions	Charge Quantity (*W*/kg)	Horizontal Distance between Charging Center and Protective Door (*S/*m)	Suspension Height of Charging Center (*h*/m)
1	1.36	11.0	0.9
2	10.2	11.0	0.9
3	20.0	7.0	0.9

**Table 2:** Material parameters of explosives.

Explosive	*ρ*_0_/(kg·m^−3^)	*A*/Pa	*B*/Pa	*R*_1_	*R*_2_	ω	Detonation Velocity *D*/(m·s^−1^)	Explosion Pressure *P*_CJ_/Pa	Internal Energy per Unit Volume *E*_0_/(J·m^−3^)
TNT	1630	3.74 × 10^11^	3.75 × 10^9^	4.15	0.9	0.35	6930	2.1 × 10^10^	6.0 × 10^9^

**Table 3:** Observation point number and position coordinates.

Number	Position Coordinates			Remarks
	X/mm	Y/mm	Z/mm	
1	18,200	0	2350	Observation points on the door frame wall: observation points 1, 2 and 3 correspond to measurement points P1, P3 and P2 in the test respectively.
2	18,200	−825	1100	
3	18,200	825	1100	
4	18,100	700	100	Observation point on the protective gate
5	18,100	700	1150	
6	18,100	700	2200	
7	18,100	0	100	
8	18,100	0	1150	
9	18,100	0	2200	

**Table 4:** Comparison between experimental and calculated values of shock wave parameters at measuring points.

Test Conditions	Observation Point	Peak Overpressure/MPa			Impulse/Pa·s		
		Test Value	Calculated Value	Error/%	Test Value	Calculated Value	Error/%
2	1	1.52	1.42	−6.6	11,727.0	12,347.4	5.3
	2	2.39	2.45	2.5	15,267.9	16,467.5	7.9
	3	2.48	2.45	−1.2	×	12,254.8	×
3	1	3.48	3.12	−10.3	×	18,445.1	×
	2	6.13	7.73	26.1	◊	25,956.7	◊
	3	◊	7.73	◊	◊	25,956.7	◊

Note: “◊” means that this data is not obtained in the test; “×” indicates that the test curve has obvious drift, and this group of data is discarded.

**Table 5:** Shock wave load at each observation point on the protective door sash.

Position	Test Point Number	Test Conditions 2		Test Conditions 3	
		Peak Overpressure/MPa	Impulse Value/Pa·s	Peak Overpressure/MPa	Impulse Value/Pa·s
Lower part	4	2.30	21,183.5	5.84	33,980.3
	7	1.51	21,056.4	4.67	33,827.7
Middle part	5	1.63	20,133.4	4.75	32,614.6
	8	1.67	20,148.5	4.93	32,641.0
Upper part	6	1.57	20,285.0	4.06	32,338.2
	9	1.34	20,292.2	3.07	32,496.2

**Table 6:** Values of strain rate parameters for different types of steels.

Material	*C*/s^−1^	*P*	literature
Mild steel	40.4	5	Cowper and Symonds [18]
Aluminium alloy	6500	4	Bodner and Symonds [19]
α-titanium (Ti50A)	120	9	Symonds and Chon [20]
304 Stainless steel	100	10	Forrestal and Sagartz [21]
High-strength steel	3200	5	Paik and Chung [22]

**Table 7:** Material parameters of finite element calculation model.

Assembly	Density/(kg/m^3^)	Poisson Ratio	Elastic Modulus/GPa	Yield Strength/MPa	Hardening Modulus/GPa	Strain Rate Parameter		Failure Strain
						C	P	
Skeleton, panel, backing plate	7800	0.3	206	235	0.21	40.4	5	0.3
Screw rod, sleeve	7800	0.3	200	355	0.21	40.4	5	0.3
Door frame	2500	0.2	32.5	-	-	-	-	-

Note: “-” indicates that the value does not exist.

**Table 8:** Peak displacement of the protective door under different calculation conditions.

Calculation Condition Number	Outer Panel	Inner Panel	Skeleton	Maximum Displacement/mm	Maximum Displacement Reduction Amplitude/%
1	Q235	Q235	Q235	97.4	0
2	Q275			96.5	0.9
3	Q345			95.8	1.6
5	Q235	Q275	Q235	70.2	27.9
6		Q345		55.7	42.8
7	Q235	Q235	Q275	95.3	2.2
8			Q345	92.7	4.8

Note: “0” indicates that the calculated value of this working condition is the reference value.

**Table 9:** Calculation condition table for the analysis of the influencing factors of geometric dimensions.

Calculation Condition Number	$h_{1}/\mathrm{mm}$	${h^{\prime }}_{1}/\mathrm{mm}$	$h_{2}/\mathrm{mm}$	$h_{3}/\mathrm{mm}$	$h_{4}/\mathrm{mm}$
1	6	8	8	6	100
2	8				
3	10				
4	12				
5	14				
6	8	6	8	6	100
7		10			
8		12			
9		14			
10	8	8	6	6	100
11			10		
12			12		
13			14		
14	8	8	8	8	100
15				10	
16				12	
17				14	
18	8	8	8	6	60
19					80
20					120
21					140

## Data Availability

Not applicable.
